# Evaluation of topical cysteamine therapy in the *CTNS*^−/−^ knockout mouse using in vivo confocal microscopy

**Published:** 2011-10-08

**Authors:** Jennifer L. Simpson, Chyong Jy Nien, Kevin J. Flynn, James V. Jester

**Affiliations:** Gavin Herbert Eye Institute, University of California Irvine, Irvine, CA

## Abstract

**Purpose:**

The purpose of this study was to assess the ability of quantitative in vivo confocal microscopy (CM) to detect changes in cystine crystal volume in the cystinosisn (*Ctns*^−/−^)mouse cornea following topical cysteamine therapy.

**Methods:**

Fifteen *Ctns*^−/−^ mice were sequentially followed using in vivo CM from 3 to 10 months of age. In a second experiment, five mice receiving topical cysteamine eyedrops (0.55%) for 4 weeks were compared to five untreated mice. The volume of corneal cystine crystals was determined by thresholding and counting high intensity pixels in the in vivo CM scans and dividing by the stromal volume to calculate a crystal volume index (CVI).

**Results:**

Corneal crystals progressively increased in density with age, reaching a peak density at 6–8 months and showing a 70 fold increase in CVI. Eyes treated with cysteamine drops showed significantly less crystal accumulation compared to control eyes (p<0.001) with only a 15% increase in treated eyes (p=ns) compared to 173% increase (p<0.04) for untreated eyes.

**Conclusions:**

Measurement of CVI shows that there is a progressive increase in cystine crystal volume up to 8 months of age and that cysteamine eyedrops significantly inhibits progression in the *Ctns*^−/−^ mouse. These findings are similar to those seen clinically in patients with cystinosis, and suggest that measurement of CVI in the *Ctns*^−/−^ mouse may be used as a model to develop novel therapeutic strategies for treating corneal cystinosis.

## Introduction

Cystinosis is an autosomal recessive disease caused by a mutation in the lysosomal membrane transport gene, cystinosin (*CTNS*) located on chromosome 17p13 [1-5]. Accumulation of cystine in lysosomes leads to formation of cystine crystals in various tissues and organs, including the kidney, muscle, thyroid, brain and eye [5-11]. Different *CTNS* mutations are associated with varying degrees of disease severity, with patients categorized into one of three severity groups based on their age of onset and symptoms [2]. The most severe form of the disease, infantile cystinosis, results from the complete lack of cystine lysosomal transport, with end-stage renal disease by age 10 [6,12].

Corneal cystine crystals appear within the first 16 months of life, increase linearly during the first decade until they plateau in early adolescence [3,4,9-11]. The non-nephropathic forms of cystinosis (juvenile and ocular cystinosis) demonstrate some residual membrane transport function and are associated with later onset and more limited systemic manifestations. However, corneal crystals are also present on these less severe forms of the disease [13,14].

Oral administration of cysteamine (HS-CH_2_-CH_2_-NH_3_) or β-mercaptoethylamine has been the mainstay of cystinosis therapy since 1994, when Cystagon^™^ was approved by the USA FDA [15-17]. Cysteamine reacts with cystine to produce the single sulfide amino acid cysteine, plus a cysteine-cysteamine mixed disulfide that exits the lysosome via the lysine transporter. By circumventing the cystinosin transporter defect [18], oral cysteamine has significantly improved overall prognosis [5,12,19,20]. However, systemic administration of cysteamine has no effect on corneal cystinosis [6,21-23] because of inadequate local cysteamine concentrations [19]. Thus, cysteamine eyedrops must be applied to the ocular surface at hourly intervals to achieve sufficient drug concentrations to reduce corneal cystine levels. While this treatment strategy is been shown partially successful, the drug dosing regimen is overly burdensome and patient compliance is poor leaving many patients to suffer from the chronic effects of corneal cystinosis.

Recently, a cystinosis (*Ctns*^−/−^) knockout mouse has been generated that shows development of cystine crystals in multiple tissues and organs, including the cornea [24-27]. Although the *Ctns*^−/−^ mouse does not under go proximal tubulopathy in the kidney, quantitative studies of organ function indicate that kidney and eye function progressively decrease with age indicating that the *Ctns*^−/−^ mouse model mimics disease progression seen clinically and is therefore a potential model to study novel therapeutic strategies for treating human cystinosis [25,28-30].

We have recently developed a quantitative method to evaluate corneal cystine crystal volume using in vivo confocal microscopy (CM) to assess progression of corneal cystinosis in the *Ctns*^−/−^ mouse model [31]. Our studies showed that cystine crystals in the mouse cornea were first detected at 3 months of age in the posterior stroma/corneal endothelium and that crystal volume progressively increased over time up to 7 months of age, followed by stromal inflammation, decreased stromal cell density and corneal scarring by 8–12 months of age. In the present study, we evaluated the sensitivity of using in vivo CM to measure temporal changes in corneal cystine crystal volume to detect the effectiveness of standard topical cysteamine therapy.

## Methods

### Mice

A total of 25 *Ctns*^−/−^ knockout mice were used in this study. Fifteen *Ctns*^−/−^ mice were followed and examined over time at each of the following time points: 3, 4, 6, 8, and 10 months, respectively. At each time point, animals were anesthetized with intraperitoneal injections of ketamine HCl (100 mg/Kg bodyweight; Bioniche Pharma, Lake Forest, IL) and xylazine (20 mg/Kg bodyweight; Lloyd Laboratories, Shenandoah, IO) and in vivo confocal microscopy (CM) was used to assess the presence and location of cystine crystals. At 10 months of age, animals were sacrificed by cervical dislocation under anesthesia.

Ten 5 month old, *Ctns*^−/−^ knockout mice were used to measure the changes in the crystal volume after treating with 0.55% cysteamine eyedrops (Leiter's Pharmacy, San Jose, CA) for 4 weeks. Briefly, 5 animals were treated with cysteamine eyedrops in both eyes, four times a day, while 5 animals were used as controls. Before and after treatment, animal corneas were scanned using in vivo CM to assess the presence and location of cystine crystals. Animals were then sacrificed by cervical dislocation after anesthesia. All procedures were approved by the UCI IACUC and conducted in accordance with ARVO Statement for the Use of Animals in Ophthalmic and Vision Research.

### In vivo confocal microscopy

Animals underwent in vivo CM scanning using a tandem scanning confocal microscope (TSCM; Tandem Scanning Corporation, Reston, VA) with a 24× surface-contact objective (numerical aperture, 0.6; working distance, 1.5 mm), encoder mike controller (Oriel 18011; Oriel, Stratford, CT) for focal plane control, and a low light level camera (MTI VE-1000; Dage MTI, Michigan City, IN). One drop of preservative-free, Refresh Tears (Allergan, Irvine, CA) was placed on the tip of the objective as a coupling gel. All camera settings were kept constant throughout the experiment. For each eye, repeated data sets were obtained from select central and peripheral corneal locations. Three to five through-focus data sets were collected around these regions for assessment of cystine crystal volume using Metamorph Image Analysis software (Molecular Devices, Downingtown, PA).

### Quantitative assessment of cystine crystal content

To measure the cystine crystal content in the cornea, through focus image data sets were analyzed by Metamorph Image Processing Software (Molecular Devices). Initially, the stromal regions were extracted from the through focus data set and then thresholded using the Threshold subroutine to include all high intensity pixels representing light scattering from the cystine crystals. Threshold regions were set to include pixels intensity from 100 to 255. Pixels within the threshold region were then counted using the *Measure* subroutine for all planes in the image stack to record the crystal volume. To calculate a *Percent Crystal volume Index* (CVI), the crystal volume was divided by the extracted stromal volume multiplied by 100.

### Statistical analysis

Each eye was considered independently and results were reported as the mean±standard deviation (SD). Differences over time and between treatment groups were assessed by two-way repeated-measures ANOVA and Bonferroni multiple comparisons (Sigma Stat version 3.11; Systat Software Inc., Point Richmond, CA).

## Results

### Progression of corneal cystinosis in the *Ctns*^−/−^ mouse

**Figure 1:**
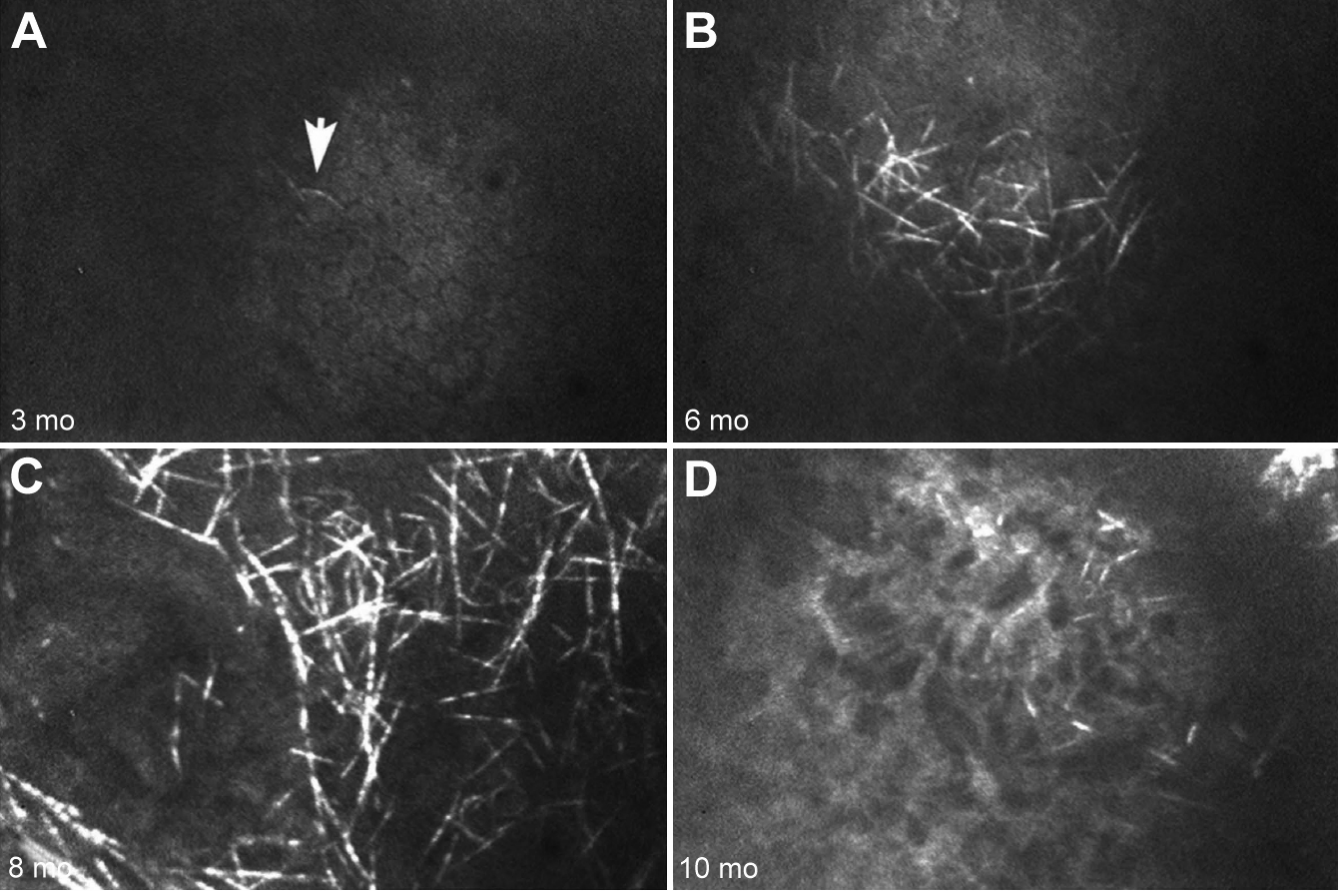
Confocal images of *Ctns*^-\-^ mouse cornea. Confocal images of the same *Ctns*^−/−^ cornea over time at 3 months (**A**), 6 months (**B**), 8 months (**C**), and 10 months of age (**D**). Each panel shows a xy (upper) slice through a 3D stack. Cystine crystals were identified as small, 20 µm long, needle-like crystals in the peripheral and central cornea. Note that cystine crystals increase progressively in quantity up to 8 months of age (**A**, **B**, and **C**), but at 10 months, the cornea scarred and showed increased opacity.

A total of 15 *Ctns*^−/−^ mice were examined and followed using serial in vivo CM. Seven animals died at different time points during the course of the study and were excluded from the analysis. Using in vivo CM, a few cystine crystals were detected at 3 months of age (Figure 1A) with increasing crystal volume up to 6 to 8 months of age (Figure 1B,C respectively). Crystals first appeared in the peripheral posterior stroma/corneal endothelium and then progressed anteriorly and centrally with age. By 10 months, *Ctns*^−/−^ mouse corneas showed breakdown of cystine crystals combined with corneal neovascularization, fibrosis, and scarring (Figure 1D).

**Figure 2:**
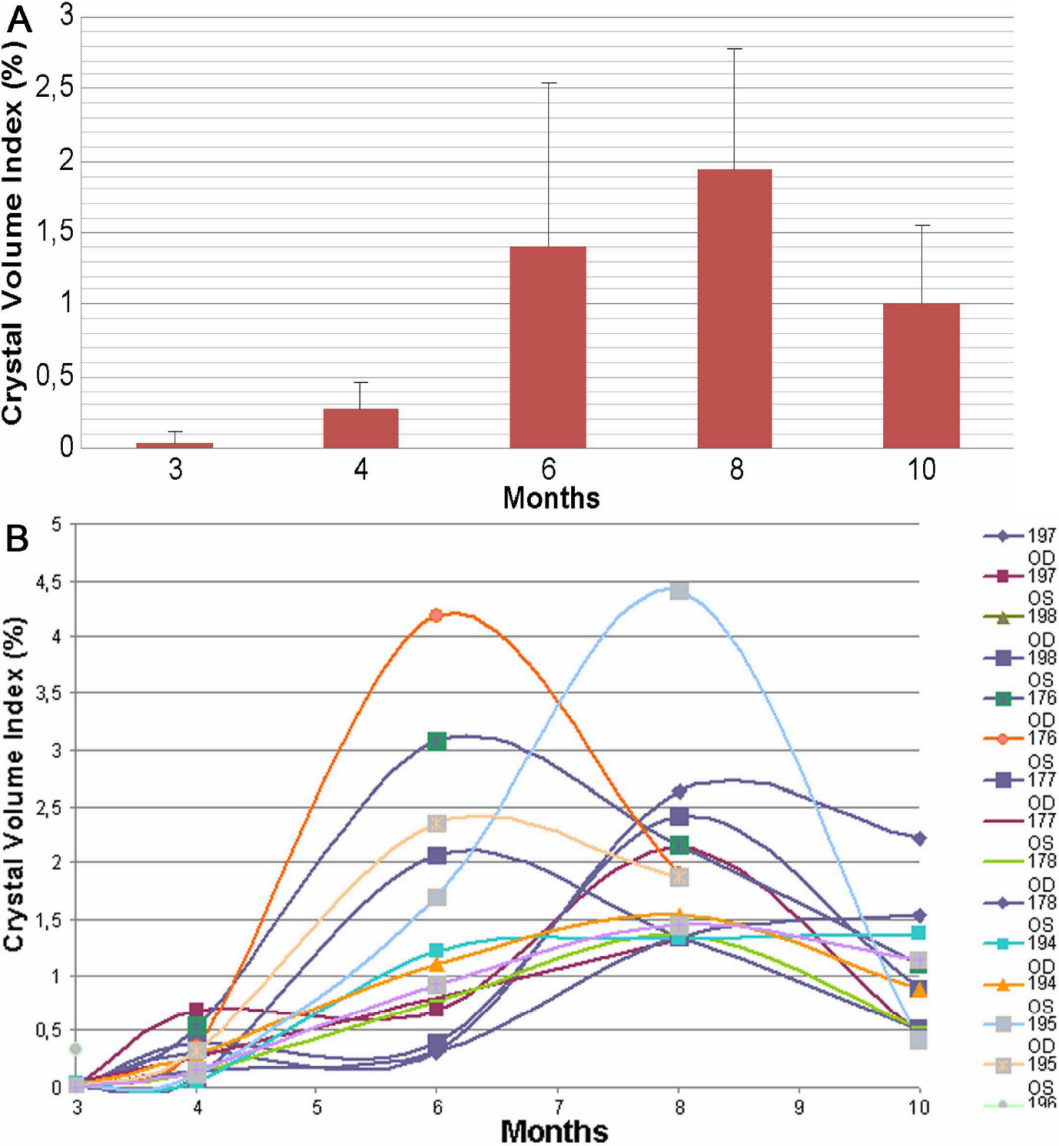
Cystine crystal quantification measured as the percentage (%) of crystals per stromal volume over time. We observed a progressive increase in the crystal content in corneas from 3 to 8 months of age. However, at 10 months of age, crystal volume appeared to decrease due to breakdown of crystals and/or scarring. **A**: The average and standard deviation values over time. **B**: All the individual eyes that were followed over 10 months.

Table 1 and Figure 2 summarize the time-course changes of the crystal volume index (CVI) of all the evaluated eyes, excluding animals removed from the study. In this group, 4 eyes reached the highest CVI at 6 months averaging 2.9%±0.94, 8 eyes reached the highest content of crystals at 8 months of age with an average CVI of 2.15%±1.04 and 2 eyes showed a progressive increase in the CVI that peaked at 10 months of age. Overall, the maximum increase in crystal deposition was from 3 to 8 months with an average 70 fold increase that was followed by decreasing volume due presumably to corneal inflammation, neovascularization and scarring.

**Table 1:** Crystal volume index (CVI) time course in study corneas.

**Eye ID**	**3 months**	**4 months**	**6 months**	**8 months**	**10 months**
197OD	0	0.31	1.31	2.64	2.22
197 OS	0	0.68	1.68	2.13	0.45
198 OD	SCAR	SCAR	SCAR	SCAR	SCAR
198 OS	0	0.38	1.38	2.41	0.88
176 OD	0	0.54	3.07	2.15	1.09
176 OS	0	0.36	4.18	1.89	SCAR
177 OD	0	0.08	2.06	1.34	0.51
177 OS	0.03	0.27	0.80	1.33	SCAR
178 OD	0	0.13	0.75	1.35	0.53
178 OS	0	0.13	0.30	1.34	1.53
194 OD	0.03	0.05	1.21	1.33	1.36
194 OS	0	0.29	1.09	1.54	0.87
195 OD	0	0.11	1.68	4.40	0.41
195 OS	0	0.32	2.35	1.86	SCAR
196 OD	0.33	SCAR	SCAR	SCAR	SCAR
196 OS	0	0.15	0.91	1.44	1.13

A total of four out of the 16 eyes developed corneal scaring by 10 months of age. Within this group, one eye was already scarred at the time of the first scan at 3 months and was not used to calculate change in crystal volume with age in this study. Of the remaining 3 eyes, one eye developed corneal scarring at 4 months and 2 eyes showed scarring at 10 month of age. These eyes were not included in the calculation of crystal volume at later time points, following the development of corneal scarring.

It is interesting to observe the asymmetry of the crystal deposition in the same animal suggesting that each eye progresses as an individual case and should be treated independently. In general, eyes showed increased crystal volume from 3 to 4 months and 4 to 6 months of age. Over 70% of eyes (10/14) showed increased crystal volume from 6 to 8 months, while only 14% of eyes (2/14) showed increased crystal volume from 8 to 10 months of age. Overall, these findings suggest that the optimal age to evaluate the effects of therapy on corneal cystine crystal formation using in vivo CM should be from 3 to 6 months of age.

### Effects of cysteamine eyedrops on corneal cystinosis

After baseline, two eyes from the untreated group and one eye from the treated group showed corneal scarring and had to be removed from the study. When comparing the remaining eyes at baseline, there was no significant difference in the CVI between untreated and treated groups, averaging 0.82% and 0.70% respectively (p=0.78; Table 2).

**Table 2:** Crystal volume index (CVI) in untreated and cysteamine treated eyes.

**Eye ID**	**CVI at baseline (Mean±SD)**	**CVI after treatment (Mean±SD)**	**% change**
**Untreated**			
246 OD	SCAR	SCAR	SCAR
246 OS	0.76±0.13	SCAR	SCAR
247 OD	0.44±0.06	SCAR	SCAR
247 OS	0.41±0.15	1.77±0.52	336
275 OD	0.25±0.06	0.40±0.07	61
275 OS	0.24±0.03	0.93±0.18	283
156 OD	SCAR	SCAR	SCAR
156 OS	3.48±0.32	3.92±0.59	12
277 OD	0.46±0.26	0.78±0.45	67
277 OS	0.53±0.27	0.67±0.39	25
Mean CVI	0.82	1.41	173%
**Cysteamine treated**			
276 OD	0.38±0.13	0.41±0.17	9
276 OS	0.77±0.40	0.60±0.19	-21
278 OD	0.43±0.09	0.49±0.08	16
278 OS	0.48±0.01	0.37±0.17	-22
279 OD	0.25±0.15	0.60±0.13	137
279 OS	0.17±0.11	0.28±0.13	65
249 OS	0.82±0.26	0.73±0.01	-10
249 OD	2.45±0.53	1.13±0.55	-53
245 OD	0.55±0.17	2.87±2.32	416
245 OS	SCAR	SCAR	SCAR
Mean CVI	0.70	0.83	15%

After 4 weeks, 2 eyes in the untreated group became scarred and could not be evaluated for crystal deposition. The remaining 6 eyes in the untreated group showed a significant (173%) increase in the CVI (0.82% versus 1.41%, p≤0.04; Table 2). By contrast, the cysteamine treated group showed only a 15% increase in the CVI (from 0.70% before to 0.83% after treatment, p=ns), significantly less than the untreated group (p<0.0001). None of the eyes in the cysteamine treated group developed scarring after treatment. These results suggest that topical cysteamine eyedrops delay the progression of corneal crystal deposition.

## Discussion

In this study, we have described a significant reduction in the clinical progression of cystine crystal deposition in the *Ctns*^−/−^ mouse cornea with topical cysteamine. While the amount of corneal crystals varied between animals, crystal deposition in the posterior cornea was generally noted at 3 months. Consistent with previous studies [28,31], a 70 fold increase in crystal volume occurred between 3 months to 8 months of age in untreated corneas, after which corneas became scarred. Topical cysteamine eyedrops (applied 4 times daily for 4 weeks beginning at 5 months of age) significantly decreased corneal crystal deposition compared to untreated *Ctns*^−/−^ mice.

This data mirrors the response to topical cysteamine in human cystinosis, where substantial clearance of cystine crystals follows prolonged periods of treatment [23,32,33]. While effective, topical cysteamine has several drawbacks, including poor formulation stability, an hourly dosing schedule and poor compliance (especially during childhood and adolescent years) [22,23,32]. To overcome these shortcomings, drug development efforts have focused on the development of formulations that reduce dosing frequency, improve compliance and quality of life for patients with corneal cystinosis of application [34,35]. These efforts can potentially be accelerated using the *Ctns*^−/−^ mouse model to screen new therapeutic approaches before costly and time-consuming human clinical trials.

## References

[1] Anikster Y, Shotelersuk V, Gahl WA (1999). CTNS mutations in patients with cystinosis.. Hum Mutat.

[2] Attard M, Jean G, Forestier L, Cherqui S, van't Hoff W, Broyer M, Antignac C, Town M (1999). Severity of phenotype in cystinosis varies with mutations in the CTNS gene: predicted effect on the model of cystinosin.. Hum Mol Genet.

[3] Kalatzis V, Cohen-Solal L, Cordier B, Frishberg Y, Kemper M, Nuutinen EM, Legrand E, Cochat P, Antignac C (2002). Identification of 14 novel CTNS mutations and characterization of seven splice site mutations associated with cystinosis.. Hum Mutat.

[4] Shotelersuk V, Larson D, Anikster Y, McDowell G, Lemons R, Bernardini I, Guo J, Thoene J, Gahl WA (1998). CTNS mutations in an American-based population of cystinosis patients.. Am J Hum Genet.

[5] Cantani A, Giardini O, D'Eufemia P (1991). The treatment of cystinosis with cysteamine.. Ann Pediatr (Paris).

[6] Cantani A, Giardini O, Ciarnella Cantani A (1983). Nephropathic cystinosis: ineffectiveness of cysteamine therapy for ocular changes.. Am J Ophthalmol.

[7] Gahl WA, Kuehl EM, Iwata F, Lindblad A, Kaiser-Kupfer MI (2000). Corneal crystals in nephropathic cystinosis: natural history and treatment with cysteamine eyedrops.. Mol Genet Metab.

[8] Gahl WA, Thoene JG, Schneider JA, O'Regan S, Kaiser-Kupfer MI, Kuwabara T (1988). NIH conference. Cystinosis: progress in a prototypic disease.. Ann Intern Med.

[9] Tsilou E, Zhou M, Gahl W, Sieving PC, Chan CC (2007). Ophthalmic manifestations and histopathology of infantile nephropathic cystinosis: report of a case and review of the literature.. Surv Ophthalmol.

[10] Tsilou ET, Rubin BI, Reed G, Caruso RC, Iwata F, Balog J, Gahl WA, Kaiser-Kupfer MI (2006). Nephropathic cystinosis: posterior segment manifestations and effects of cysteamine therapy.. Ophthalmology.

[11] Tsilou ET, Rubin BI, Reed GF, Iwata F, Gahl W, Kaiser-Kupfer MI (2002). Age-related prevalence of anterior segment complications in patients with infantile nephropathic cystinosis.. Cornea.

[12] Gahl WA, Reed GF, Thoene JG, Schulman JD, Rizzo WB, Jonas AJ, Denman DW, Schlesselman JJ, Corden BJ, Schneider JA (1987). Cysteamine therapy for children with nephropathic cystinosis.. N Engl J Med.

[13] Gahl WA, Thoene JG, Schneider JA (2002). Cystinosis.. N Engl J Med.

[14] Iwata F, Kaiser-Kupfer MI (1994). Ocular manifestations of metabolic disorders.. Curr Opin Ophthalmol.

[15] Gahl WA (2003). Early oral cysteamine therapy for nephropathic cystinosis.. Eur J Pediatr.

[16] Gahl WA, Balog JZ, Kleta R (2007). Nephropathic cystinosis in adults: natural history and effects of oral cysteamine therapy.. Ann Intern Med.

[17] Kleta R, Gahl WA (2004). Pharmacological treatment of nephropathic cystinosis with cysteamine.. Expert Opin Pharmacother.

[18] Thoene JG, Oshima RG, Crawhall JC, Olson DL, Schneider JA (1976). Cystinosis. Intracellular cystine depletion by aminothiols in vitro and in vivo.. J Clin Invest.

[19] Tsilou ET, Thompson D, Lindblad AS, Reed GF, Rubin B, Gahl W, Thoene J, Del Monte M, Schneider JA, Granet DB, Kaiser-Kupfer MI (2003). A multicentre randomised double masked clinical trial of a new formulation of topical cysteamine for the treatment of corneal cystine crystals in cystinosis.. Br J Ophthalmol.

[20] Markello TC, Bernardini IM, Gahl WA (1993). Improved renal function in children with cystinosis treated with cysteamine.. N Engl J Med.

[21] Hsuan JD, Harding JJ, Bron AJ (1996). The penetration of topical cysteamine into the human eye.. J Ocul Pharmacol Ther.

[22] Jones NP, Postlethwaite RJ, Noble JL (1991). Clearance of corneal crystals in nephropathic cystinosis by topical cysteamine 0.5%.. Br J Ophthalmol.

[23] Kaiser-Kupfer MI, Fujikawa L, Kuwabara T, Jain S, Gahl WA (1987). Removal of corneal crystals by topical cysteamine in nephropathic cystinosis.. N Engl J Med.

[24] Cherqui S, Kalatzis V, Forestier L, Poras I, Antignac C (2000). Identification and characterisation of the murine homologue of the gene responsible for cystinosis, Ctns.. BMC Genomics.

[25] Cherqui S, Sevin C, Hamard G, Kalatzis V, Sich M, Pequignot MO, Gogat K, Abitbol M, Broyer M, Gubler MC, Antignac C (2002). Intralysosomal cystine accumulation in mice lacking cystinosin, the protein defective in cystinosis.. Mol Cell Biol.

[26] Forestier L, Jean G, Attard M, Cherqui S, Lewis C, van't Hoff W, Broyer M, Town M, Antignac C (1999). Molecular characterization of CTNS deletions in nephropathic cystinosis: development of a PCR-based detection assay.. Am J Hum Genet.

[27] Kalatzis V, Cherqui S, Antignac C, Gasnier B (2001). Cystinosin, the protein defective in cystinosis, is a H(+)-driven lysosomal cystine transporter.. EMBO J.

[28] Kalatzis V, Serratrice N, Hippert C, Payet O, Arndt C, Cazevieille C, Maurice T, Hamel C, Malecaze F, Antignac C, Muller A, Kremer EJ (2007). The ocular anomalies in a cystinosis animal model mimic disease pathogenesis.. Pediatr Res.

[29] Kalatzis V, Cherqui S, Jean G, Cordier B, Cochat P, Broyer M, Antignac C (2001). Characterization of a putative founder mutation that accounts for the high incidence of cystinosis in Brittany.. J Am Soc Nephrol.

[30] Kalatzis V, Nevo N, Cherqui S, Gasnier B, Antignac C (2004). Molecular pathogenesis of cystinosis: effect of CTNS mutations on the transport activity and subcellular localization of cystinosin.. Hum Mol Genet.

[31] Simpson J, Nien CJ, Flynn KJ, Cherqui S, Jester JV (2011). Quantitative in vivo and ex vivo confocal microscopy analysis of corneal cystine crystals in the Ctns−/− knockout mouse.. Mol Vis.

[32] Kaiser-Kupfer MI, Gazzo MA, Datiles MB, Caruso RC, Kuehl EM, Gahl WA (1990). A randomized placebo-controlled trial of cysteamine eye drops in nephropathic cystinosis.. Arch Ophthalmol.

[33] MacDonald IM, Noel LP, Mintsioulis G, Clarke WN (1990). The effect of topical cysteamine drops on reducing crystal formation within the cornea of patients affected by nephropathic cystinosis.. J Pediatr Ophthalmol Strabismus.

[34] Bozdağ S, Gumus K, Gumus O, Unlu N (2008). Formulation and in vitro evaluation of cysteamine hydrochloride viscous solutions for the treatment of corneal cystinosis.. Eur J Pharm Biopharm.

[35] Buchan B, Kay G, Heneghan A, Matthews KH, Cairns D (2010). Gel formulations for treatment of the ophthalmic complications in cystinosis.. Int J Pharm.

